# Pertussis Circulation Has Increased T-Cell Immunity during Childhood More than a Second Acellular Booster Vaccination in Dutch Children 9 Years of Age

**DOI:** 10.1371/journal.pone.0041928

**Published:** 2012-07-31

**Authors:** Rose-Minke Schure, Lia de Rond, Kemal Öztürk, Lotte Hendrikx, Elisabeth Sanders, Guy Berbers, Anne-Marie Buisman

**Affiliations:** 1 Laboratory for Infectious Disease and Perinatal Screening, Center for Infectious Diseases Control, National Institute for Public Health, Bilthoven, the Netherlands; 2 Department of Pediatric Immunology, University Medical Center/Wilhelmina Kinder Ziekenhuis, Utrecht, the Netherlands; University of Pittsburgh, United States of America

## Abstract

Here we report the first evaluation of T-cell responses upon a second acellular pertussis booster vaccination in Dutch children at 9 years of age, 5 years after a preschool booster vaccination. Blood samples of children 9 years of age were studied longitudinally until 1 year after the second aP booster and compared with those after the first aP booster in children 4 and 6 years of age from a cross-sectional study. After stimulation with pertussis-vaccine antigens, Th1, Th2 and Th17 cytokine responses were measured and effector memory cells (CCR7-CD45RA-) were characterized by 8-colour FACS analysis. The second aP booster vaccination at pre-adolescent age in wP primed individuals did increase pertussis-specific Th1 and Th2 cytokine responses. Noticeably, almost all T-cell responses had increased with age and were already high before the booster vaccination at 9 years of age. The enhancement of T-cell immunity during the 5 year following the booster at 4 years of age is probably caused by natural boosting due to the a high circulation of pertussis. However, the incidence of pertussis is high in adolescents and adults who have only received the Dutch wP vaccine during infancy and no booster at 4 years of age. Therefore, an aP booster vaccination at adolescence or later in these populations might improve long-term immunity against pertussis and reduce the transmission to the vulnerable newborns.

**Trial Registration:**

Controlled-Trials.com ISRCTN64117538

## Introduction

Since the introduction of pertussis vaccination in the developed world, disease incidence, morbidity and mortality have decreased [1]. Over the last two decades, however, pertussis has reemerged [2]. In the Netherlands rises in pertussis incidence are seen every 2–3 years from 1996 onwards [3]. For this reason, several changes in the Dutch pertussis immunization program have been implemented. In 1999, primary vaccinations with the whole cell (wP) vaccine were advanced to 2, 3 and 4 months of age, followed by a booster at 11 months of age. In 2001, a high-dose acellular pertussis (aP) vaccination was introduced at 4 years of age as a preschool booster. Additionally, in 2005 the wP-component was replaced by an aP-component in the DTP-IPV-Hib primary schedule for infants in the first year of life. As a result, the previous pertussis peak-incidence in the age-cohort of children of 4–5 years in 2001 has shifted towards the age-cohort of 12–13 years nowadays in the Netherlands [4] (F.R. Mooi, personal communication). In general, the burden of whooping cough has shifted from young children to pre-adolescents and adults [5].

The primary sources of infection of the unvaccinated or not fully vaccinated neonates who have the highest risk for both severe symptoms of disease and pertussis-related deaths [6] appear to be household contacts, like mothers and siblings [7]. A number of countries have therefore implemented an acellular pertussis booster vaccination in adolescents and young adults, also hoping to protect infants via herd effects [8].

**Figure 1:**
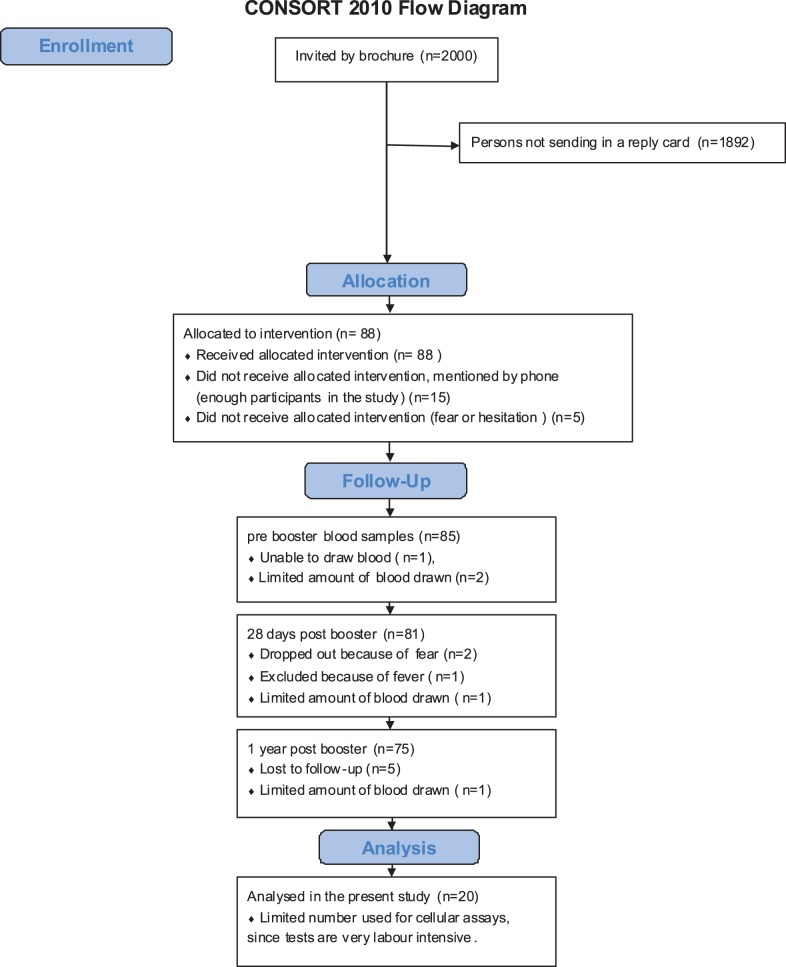
Consort 2010 Flow Diagram. Study participants, during the recruitment of children 9 years of age who received *Boostrix-IPV*™ as a second aP booster vaccination.

The mechanism of immunity to pertussis involves a range of both humoral and cellular immune responses, directed at several pertussis antigens included in the vaccines [9]. Although antibody levels wane relatively fast after both natural infection and vaccination, a certain level of protection via cell-mediated immunity seems to persist [10], [11]. We recently demonstrated that an extra aP booster vaccination at 9 years of age induced elevated antibody responses that persisted even after one year due to enhanced memory B-cell levels one month post booster [12]. However, data on pertussis-specific T-cell immunity in response to a pre-adolescent booster vaccination are lacking, though T-cells are suggested to be relevant for clinical protection [13].

The aim of this study was to investigate longitudinal T-cell immunity before and after a pre-adolescent aP booster vaccination at 9 years of age. Pertussis-specific T-cell immunity was measured before, 1 month and 1 year after booster vaccination. For comparison, T-cell immunity was evaluated in 4- and 6-years old children. All children had previously been vaccinated at infant age with four wP vaccinations and had received an acellular pertussis booster vaccine at 4 years of age.

**Figure 2:**
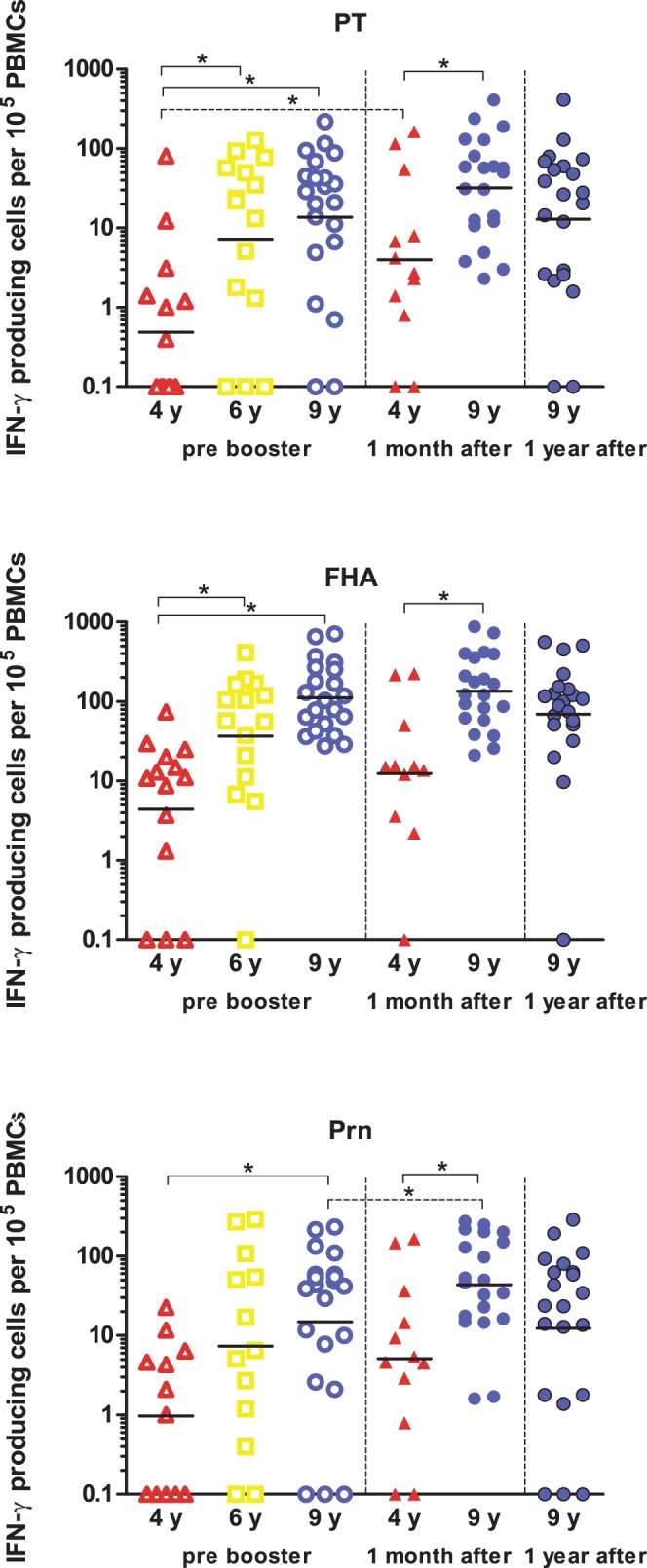
Numbers of IFN-γ producing cells. PBMCs of children 4 years of age (red open triangles) (n = 14), 6 years of age (yellow open squares) (n = 15) and 9 years of age (blue open circles) (n = 20) pre-booster have been stimulated with PT, FHA or Prn for 5 days and subsequently numbers of IFN-γ producing cells have been determined. Children 9 years of age have been studied longitudinally at 1 month (blue closed circles) and 1 year (dark blue filled circles) post a second aP booster vaccine (n = 20) and children 4 years of age have been studied cross-sectionally at 1 month post a first aP booster vaccine (red filled triangles) (n = 11). Horizontal lines represent geometric means of IFN-γ producing cells per 100.000 stimulated PBMCs. * =  significant difference between groups.

## Materials and Methods

### Subjects and Study Design

In this study (ISRCTN64117538), blood samples were collected to evaluate pertussis booster vaccination in 20 children aged 9 years as described in the Consort 2010 Flow Diagram (Fig. 1). The protocol for this trial and supporting CONSORT checklist are available as supporting information; see Checklist S1 and Protocol S1. Blood samples (15 ml) were collected before, at 1 month and at 1 year post booster aP vaccination. Data were compared with those obtained from children aged 4 and 6 years (n = 15) who had participated in a cross-sectional observational study in the Netherlands (ISRCTN65428640), in 2007–2008 [14], [15]. From children of 4 years of age, PBMCs prebooster (n = 15) as well as those at 28 days after booster (n = 12) were available.

These studies were conducted according to the Declaration of Helsinki, Good Clinical Practice Guidelines with the approval of the relevant ethics review committee (Medisch Ethische Toestingscommissie (METC) in Almere, the Netherlands and the Central Committee on Research Involving Human Subjects (CCMO), the Hague, the Netherlands). Written informed consent was obtained from both parents or legal representatives. In both studies, sexes were equally distributed.

**Figure 3:**
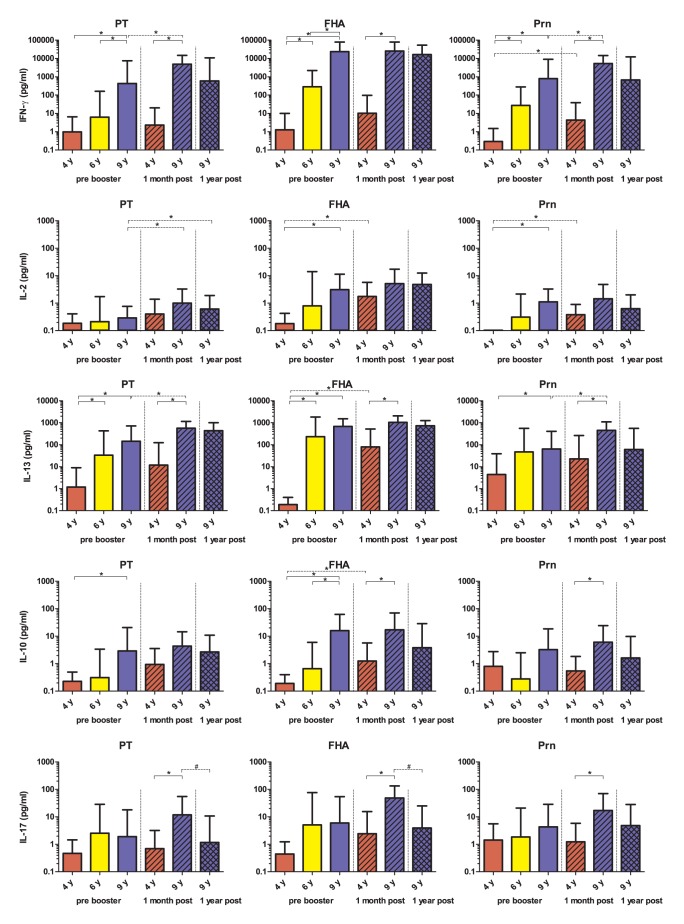
Pertussis protein-specific cytokine responses. Th1, Th2 and Th17 and IL-10 responses in supernatants of PT, FHA and Prn stimulated PBMCs of children of 4 years of age (red bars), 6 years of age (yellow bars) and 9 years of age (blue bars) are presented as GMCs with 95% confidence intervals. Additionally, cytokine responses of children 4 years of age at 1 month post a first aP booster vaccine (red hatched bars) and children 9 years of age at 1 month (blue hatched bars) and 1 year (blue cross-hatched bars) post a second aP booster vaccine are shown. * =  significant increase between groups # =  significant decrease between groups.

### Vaccines

The 9 years old children received *Boostrix-IPV*™ (GlaxoSmithKline Biologicals S.A., Rixensart, Belgium) containing 8 µg pertussis toxin (PT), 8 µg filamentous heamagglutinin (FHA) and 2.5 µg pertactin (Prn) for the pertussis antigens. All children of 4, 6 and 9 years of age received DTwP-IPV + Hib (NVI, Bilthoven, the Netherlands) at 2, 3, 4 and 11 months of infant age. At 4 years of age a high-dose booster *ACV-SB*™ containing 25 µg PT, 25 µg FHA and 8 µg Prn was administered according to the Dutch NIP.

### Cell Stimulation and IFN-γ ELISPOT

PBMCs were isolated for measurement of IFN-γ spots per 100,000 PBMCs directed against the 3 *B. pertussis* vaccine antigens included in the booster vaccine. Cells were stimulated with 2 µg/ml PT (inactivated for 10 minutes at 80°C) or FHA (Kaketsuken, Kumamoto, Japan) and 4 µg/ml of recombinant Prn [16] as previously tested to be the optimal antigen concentrations to stimulate PBMCs to produce cytokines. As positive controls 5 µg/ml pokeweed mitogen (PWM) (Sigma Chemicals, St. Louis, Mo.) and 150 Lf/ml tetanus toxoid (Td) (NVI, Bilthoven, the Netherlands) were used. Non-stimulated (NS) cells served as negative controls. B-cells were removed by anti-CD19 coupled magnetic beads and used for B-cell stimulations [14]. PBMCs depleted of CD19+ cells were counted and 300,000 cells were cultured for 5 days incubated at 37°C and 5% CO_2_. Preliminary experiments indicated similar results in cytokine production at day 5 from PBMCs and PBMCs depleted from B-cells, both stimulated with pertussis and tetanus antigens. At day 5, all cells were harvested and incubated on 96-well anti-IFN-γ (Mabtech, Nacka Strand, Sweden) coated plates (Millipore MSIP4510, Danvers, Ma.). The next day, plates were developed by anti-IFN-γ-biotin (Mabtech), extravidin (Sigma) and BCIP/NBT. After each development step the plates were washed. Dried plates were analysed and spot numbers assessed by automatic computer-assisted ImmunoScanPro reader (CTL Europe, Bonn, Germany).

### Multiplex Assay

Cell culture supernatants were collected after 24 hours for IL-2 and after 5 days for IL-10, IL-13, IFN-γ and IL-17 and frozen at −80°C until further use. Cytokine concentrations were measured with the Bioplex pro human cytokine plex (Bio-rad, Hercules, CA, USA) according to manufacturer’s protocol, utilizing standard curve concentrations and no sample dilution. Bioplex validation kits were used to calibrate Bioplex systems (Bio-rad).

**Table 1:** Th1 and Th2 cytokine responses in children of 9 years of age.

		PT			FHA			Prn		
Cytokine	Blood sampling	n	GMC	95% CI	n	GMC	95% CI	n	GMC	95% CI
IFN-γ	Pre booster	17	429	24–7538	17	23555	7003–79231	17	790.1	70–8965
IFN-γ	+1 month	17	4883*	1620–14713	17	25725	8426–78540	17	5267 *	1932–14359
IFN-γ	+1 year	17	601	33–10911	17	16402	4964–54190	17	673	37–12387
IL-13	Pre booster	17	146	30–717	17	685	307–1532	17	64	10–410
IL-13	+1 month	17	573*	282–1165	17	1055	536–2075	17	451*	186–1096
IL-13	+1 year	17	439	192–1003	17	730	419–1269	17	61	7–558

* =  Significantly higher post booster compared to pre-booster p>0.05.

### 8-colour FACS Analysis

To measure proliferation of cells, one million PBMCs, not depleted from B-cells, were stained with 5 µM CFSE for 10 minutes in the dark at 4°C and stimulated for 5 days with 5 µg/ml PT or 10 µg/ml FHA (Novartis Siena Italy, kindly donated by Dr. Clara Ausiello) or 4 µg/ml Prn [16]; not-stimulated cells (NS) served as controls. In preliminary experiments within an international collaboration, the optimal antigen concentrations for FACS analysis have been tested elsewhere [17]. Moreover, the PT and FHA antigens provided by Novartis and purchased from Kaketsuken as described for the IFN-γ ELIspot-assay have been compared and showed similar results. At day 5 golgiplug (BD Biosciences, San José, USA) was added to block intracellular transport processes before further intracellular cytokines staining. Cells were collected, washed and stained for life-dead-staining by Aqua (Invitrogen, Paisley, Scotland, UK) and the cell surface markers APC-7-labelled CD4 (BD), PE-Cy7-labelled CD45RA (BD), and PE-labelled CCR7 (R&D systems, Minneapolis, USA). Subsequently, cells were resuspended in Cytofix/Cytoperm Plus kit (BD) and stained for V450-labelled CD3 (BD), PerCP-Cy5.5 labelled TNF-α (Biolegend, San Diego, CA, USA) and APC labelled IFN-γ (BD). After washing, the cells were analysed using the FACS canto cytometer (BD) in combination with Diva software (version 5.2 BD) and FlowJo software (Mac-version 9.0.2, Treestar US, Ashland, OR). Proliferated (CFSE-) viable T-helper-cell populations (CD3+CD4+) were divided in central memory (CCR7+CD45RA-) and effector memory T-cell (CCR7-CD45RA-) populations and analysed for Th1 cytokine production as described recently (17). CD3+CD4- cells were considered to be CD8+ cells.

### Flow Cytometric Data Analysis

Flow Cytometry Standard format 3.0 files were exported and data were evaluated using FlowJo software. Dead cells were excluded if stained with Aqua amine-reactive dye. Lymphocytes were gated based on SSC/FSC characteristics. Using FSC-A and FSC-H, singlets were gated on, based on CD3 and CD4 staining, CD3+CD4+ cells were gated within the viable lymphocyte singlet gate.

**Figure 4:**
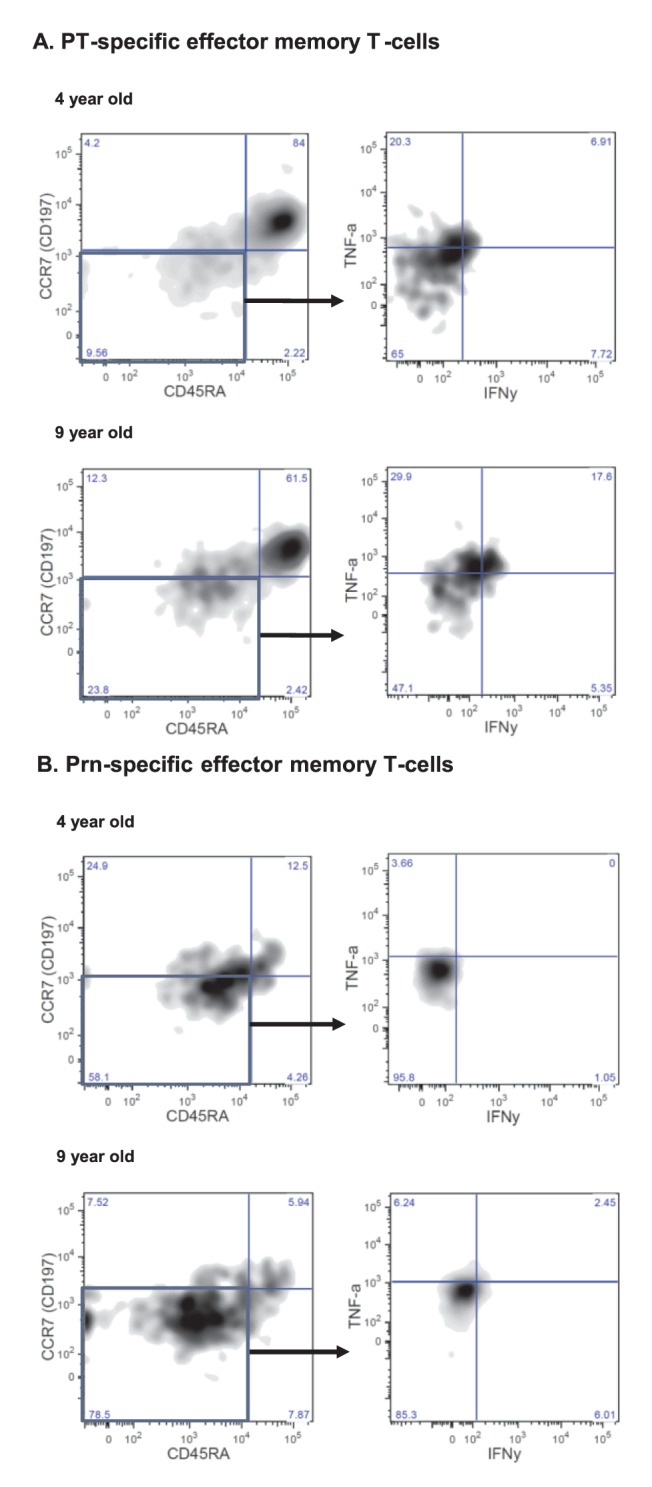
Flow-cytometry analysis of pertussis-specific CD3+CD4+ T-cells. PBMCs of children 4 and 9 years of age pre-booster were stimulated with PT and Prn for 5 days and analyzed by 8-colour FACS analysis. T-cells which have proliferated upon stimulation (CFSE-) were characterized phenotypically by CD45RA and CCR7 and the effector memory cells (CD45RA- and CCR7-) were further analyzed functionally (IFN-γ+ and TNF-α+). The results of a representative child of 4 years and 9 years of age specific for PT (A) and Prn (B) are presented.

### Statistical Methods

Cytokine concentrations and IFN-γ spots per 100,000 PBMCs were measured against the different pertussis vaccine antigens and geometric mean concentrations (GMCs) and 95% confidence intervals (CI) of PT-, FHA-, Prn-specific T-cell cytokines were calculated. Cytokine concentrations and numbers of IFN-γ producing cells in the different groups were compared with the Mann-Whitney-Wilcoxon signed-rank test. For the longitudinal samples of the 9 years old children, the Wilcoxon-matched-pairs signed rank test was performed. Moreover, we analysed the number of IFN-γ producing cells with a random effects regression model to evaluate the effect of the booster vaccination (9 years of age, longitudinally) and the effect of age (children 4, 6 and 9 years of age, cross-sectionally) separately. We used parametric repeated measurements in this model (R package version 3.1–103 R Foundation for Statistical Computing, Vienna, Austria). Significant differences between groups were found when p<0.05.

**Figure 5:**
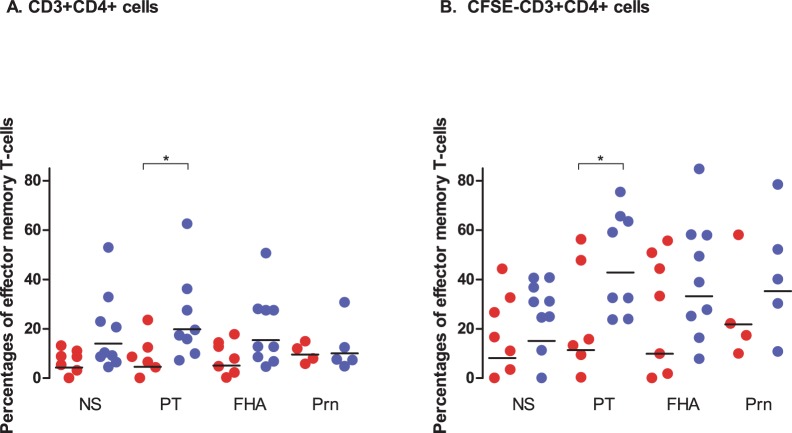
Effector memory T-cell responses. Percentages of effector memory T-cells (CD45RA-,CCR7-) in children of 4 years (red filled circles) and 9 years of age (blue filled circles) found in CD3+CD4+ T-cells (A) and proliferated (CFSE-) CD3+CD4+ T-cells (B) upon stimulation with the pertussis antigens and in non-stimulated cells (NS) Horizontal lines represent geomean values. • =  significant difference between children of 4 and 9 years of age.

## Results

### The Number of Pertussis-specific IFN-γ Producing Cells in Vaccinated Children

The number of the IFN-γ producing cells specific for PT, FHA and Prn in children 4, 6 and 9 years of age are represented in figure 2. The second aP booster vaccination at 9 years of age did not increase the numbers of pertussis specific IFN-γ producing cells. However, in general, the number of IFN-γ producing cells in these children increased with age and children aged 9 years showed significantly higher numbers of PT-, FHA- and Prn-specific IFN-γ producing cells than children at 4 years of age. Analysis with the regression model confirmed that this age-effect resulted in significantly increased numbers of IFN-γ producing cells specific for all three pertussis proteins (PT p = 0.001, FHA p = 0.0001, Prn p = 0.0072). Also, children 6 years of age showed significantly higher numbers of PT- and FHA-specific IFN-γ producing cells as compared to those of 4 years of age. Furthermore, 1 month after the second booster vaccination at 9 years of age, the geomean values of IFN-γ producing cells were higher than those at 1 month after the first booster vaccination at 4 years.

The numbers of IFN-γ producing cells had increased only slightly during a month after the second aP booster at 9 years and a significant increase was only found for Prn-specific cells. Nevertheless, only a minimal decrease after one year was observed, indicating persistently high levels of T-cell immunity both before and after the booster at 9 years of age. However, with the regression model an significantly increased booster response irrespective of age was found not only for the numbers of Prn-specific IFN-γ producing cells but also for PT at 1 month postbooster (p = 0.037 and p = 0.026, respectively).

### Cytokine Profiles in 4, 6 and 9 Years Old Pertussis Vaccine Recipients

In the same groups of children, Th1 (IFN-γ and IL-2), Th2 (IL-13), Th17 (IL-17) and regulatory T-cell (IL-10) responses specific for PT, FHA and Prn (Fig. 3) have been measured. In general, the *B. pertussis*-specific T-cell cytokine concentrations increased significantly with age already before the booster vaccinations and independently of the stimulatory antigen; all pertussis-specific Th1 and Th2 and IL-10 responses were higher at 9 years compared with children of 4 of age, except for PT-specific IL-2 and Prn-specific IL-10. Furthermore, FHA- and Prn-specific IFN-γ as well as PT- and FHA-specific IL-13 concentrations were significantly higher in children aged 6 as compared to 4 years of age. Additionally, at 9 years of age PT- and FHA-specific IFN-γ and FHA-specific IL-10 GMCs were higher than at 6 years of age.

### Effect Booster Vaccination in 9 Years Old Children

In general, at 9 years of age, prebooster IFN-γ, IL-13, IL-10 and IL-17 responses were already elevated, and the effect of the booster vaccination was mostly minimal. Nonetheless, the PT- and Prn-specific IFN-γ, as well as IL-13, and PT-specific IL-2 significantly increased 1 month after booster compared to prebooster values also at 9 years (Table 1, Fig. 3). Most cytokine responses tended to decrease again one year after the booster, but this was significant only for FHA-specific IL-17 (Fig. 3).

### Cytokine Profile after Booster Compared in 4 Years Old and 9 Years Old Children

We also compared the cytokine profiles in children of 9 years of age one month after the second aP booster vaccination with the values one month after the first aP booster in children of 4 years of age (Fig. 3). In general, the effect of the first booster vaccination at 4 years was higher than the effect upon the second booster at 9 years of age, as illustrated by significantly increased production of FHA-specific IL-2, IL-13 and IL-10 and Prn-specific Th1 responses after the first booster in children 4 years of age. Nevertheless, because of the high pre-booster levels at 9 years of age, all cytokine concentrations 1 month after the booster were significantly higher than the levels in the children at 4 years except for IL-2 and PT-specific IL-10.

### Characterization of CD3+CD4+ T-cells Comparing 4 and 9 Years Old Children

We found differences in the phenotypes of T-cells (CD3+CD4+) when children of 4 and of 9 years of age were compared upon stimulation with PT and Prn, as illustrated for one child per age-group. The T-cells were characterized by their proliferation capacity (CFSE-), expression of CCR7 and CD45RA and the production of the Th1 cytokines IFN-γ and TNF-α (Fig. 4). Several children 9 years of age showed higher percentages of pertussis-specific effector memory T-cells (CCR7-CD45RA-), and showed more Th1-cytokine production in this T-cell subpopulation (Fig. 4). Also, significantly higher percentages of PT-specific effector memory T-cells (CCR7-CD45RA-) were present in 9 years old children in the total CD3+CD4+ T-cells and also the proliferated T-cells (CFSE-, CD3+CD4+) (geomean (GM) 20% and 43%, respectively) as compared to those in 4 years old children (GM 5% and 11%, respectively) (Fig. 5). In contrast, these two age groups exhibited similar percentages of FHA- and Prn-specific effector memory T-cells (Fig. 5A). They differed in that the percentages of effector memory T-cells that proliferated (CD3+CD4+CFSE-) upon stimulation with FHA and Prn were higher only in some of the children at 9 years of age and not among the 4 year olds (Fig. 5B).

The percentages of effector memory T-cells in children 9 years of age and those 4 years of age producing Th1 cytokines, appeared comparable by both CD3+CD+ T-cells as well as proliferated CD3+CD4+CFSE- T-cells, (GM about 1.5 to 4% and GM 0.3 to 16%, respectively) (data not shown). Notably, in both groups of children effector memory cells producing both IFN-γ and TNF-α were found (GM 0.01% to 2.6%), although the percentages were low (Fig. 4).

## Discussion

The present study represents the first reported evaluation of T-cell responses upon a second acellular pertussis booster vaccination at the pre-adolescent age, 9 years of age, and 5 years after a preschool booster vaccination in children 4 years of age. The second aP booster vaccination at pre-adolescent age in wP primed individuals did increase pertussis-specific Th1 and Th2 cytokine responses. However, almost all T-cell responses were already high before the booster vaccination at 9 years of age and had enhanced significantly during the 5-year period between the two booster vaccinations. Higher T-cell booster responses were also observed 1 month after the extra booster vaccination at 9 years of age than 1 month after the preschool booster vaccination at 4 years of age. The fact that T-cell responses at 9 years of age were higher than those directly after the preschool booster at 4 years and at 6 years of age implies that natural boosting has stimulated these T-cell responses, which can be explained by the high circulation of pertussis in the Netherlands [5]. This seems in line with the presence of higher numbers of PT-specific (proliferated) effector memory CD4+ T-cells in children 9 years compared to those at 4 years of age.

In the Netherlands, an increased, high circulation of pertussis was found in the last decade as shown by carefully monitoring the incidence of pertussis as well as the evaluation of pertussis IgG levels in persons from 0 till 80 years of age in two large cross-sectional serosurveillance studies performed in 1995/6 and 2006/7 [3]. Also, the incidence of pertussis has shifted from young to older children since the implementation of the preschool aP booster vaccination at 4 years of age [4]. Very recent data have shown that the positive effect of the preschool booster lasts until the age of 13. Therefore, nowadays the increased incidence of whooping cough is mainly found in adolescents and adults [5].

It is difficult to compare our study with other studies on cell-mediated immune responses against pertussis and the effects of booster vaccinations, because each study used different age groups of children, vaccination schedules or measured different T-cell parameters [11], [18]–[22]. In contrast to other studies [18], [23], we have shown just a small increase of pertussis-specific T-cell immunity induced by a second booster at pre-adolescent age, because T-cell immunity was already elevated in these 9 years old children. The limited increase and the discrepancy with other publications describing a longer persistence of cell-mediated immune responses after booster vaccination [11], [20], [24] can be explained by the effect of a preschool booster vaccination 5 years earlier, at 4 years of age, in combination with boosting by natural infection in our study cohort.

Our results additionally showed higher numbers of pertussis-specific CD3+CD4+ effector memory T-cells in 9 years old children compared with 4 years old children. Although the percentages of effector memory T-cells producing Th1 cytokines between 4 and 9 years old children were similar, the higher number of these cells in older children resulted into higher amounts of Th1 cytokines. Notably, in both groups of children, CD3+CD4+ effector memory cells producing simultaneously IFN-γ and TNF-α upon stimulation with pertussis antigens were found, indicating that one cell is able to produce more than 1 cytokine [25].

The concentrations of T-cell cytokines in 9 years old children both before and at 1 month after booster vaccination predominantly showed Th1 IFN-γ responses and fewer IL-13 responses, associated with Th2 cytokine lineage, or IL-17 responses. Therefore, particularly in children, Th1 responses might play an important role in protection against clinical pertussis infection, while in general IL-17 was suggested to play a more prominent role in older individuals [26].

Phenotypical characterization of T-cells in adolescents by Rieber et al. after an aP booster following wP or aP priming at infancy confirmed a predominant Th1 response especially by activation of CD8 T-cells [13]. However, in contrast to these results, we found a higher percentage of CD4 effector memory T-cells (CCR7+CD45RA-) than CD8 effector memory cells in the children in our study. Next to pertussis-specific CD8+ memory T cells that may contribute to protection against clinical pertussis [13], we believe that also CD4+ T cells producing Th1-cytokines may play an important role in protection of children against clinical pertussis.

We recently showed increased pertussis-specific memory B-cell immune responses after the second aP booster vaccination in Dutch wP primed children 9 years of age that sustained at least for one year [12]. The correlation between the high numbers of pertussis-specific memory B-cells at one month after this second booster vaccination with the corresponding antibody responses still present after one year indicates the important role of the memory B-cell pool in the maintenance of antibody levels. A second aP booster vaccine affects B-cell memory more than the T-cell memory responses. We speculate that a certain level of T-helper-cell memory immunity is needed to be able to increase the B-cell responses upon booster vaccination. Epidemiological data reveal an improved protection against pertussis after the implementation of aP booster vaccines at 4 years of age [4], [5]. These studies together with the increased T-cell and B-cell responses upon the second aP-booster vaccinations at pre-adolescent age, which is strengthened by the circulation of pertussis, might result into a better protection against pertussis during adolescence. For a better understanding of the duration of pertussis-specific memory immunity, this needs to be monitored over the longer term.

Although other West European countries had already switched from wP to aP infant vaccinations in the 1990s, pertussis still circulates among these aP primed populations too and notable pertussis incidences have been equally observed in these countries as in The Netherlands that implemented the vaccine switch only in 2005. Meanwhile, concerns about the efficacy of repetitive aP booster vaccinations have risen [27]. In Canada, low vaccination coverage was observed 5 years after the implementation of adult booster vaccinations [28]. Moreover, repeated booster vaccinations in adults in general are not considered cost-effective [29], since the yet unvaccinated newborns are particularly the risk group susceptible for severe infection. As transmission studies have shown, mothers are one of the main sources of infection for the young infant [7], [30]. Thus although natural boosting of the population will also provide pertussis-specific immune responses that improve protection, the vulnerable unprotected young infant is put at risk by a high incidence of pertussis in the adult population. This means that selective vaccination of those adults who are in close contact with infants, the so-called cocooning strategy, will better reduce transmission to infants and will probably be more (cost)-effective [29], [30].

Because a majority of the Dutch population born before 1997 has only received the Dutch wP vaccine during infancy, we believe that aP booster vaccination in this part of the population will improve long-term immunity against pertussis substantially. Such a vaccine strategy might enlarge also the antibody levels in woman at childbearing age, in turn increasing the maternal transfer of antibodies to newborns who subsequently will be better protected. However, pertussis booster vaccinations in adults are expensive and the vaccination coverage in the adult population has proven to be generally low [31], [32]. Therefore, a good information and communication strategy must accompany such a booster vaccination in order to make its implementation in the late adolescent or young adult population successfully.

In conclusion, we demonstrated, in Dutch wP primed children 9 years of age, an enhanced Th1 memory immune response upon a second aP booster vaccination. These results, together with enhanced memory B-cell responses upon booster vaccination support the introduction of an aP booster vaccination for preadolescents. The positive effect of the preschool aP booster vaccination at age 4 years in combination with natural boosting of the immune responses by circulation of pertussis will probably protect wP-primed children until teenage. An aP booster vaccination at adolescence or later might improve long-term immunity against pertussis and reduce the transmission to the vulnerable newborns.

## Supporting Information

Protocol S1 **Study protocol.**
(DOC)

Checklist S1 **CONSORT checklist.**
(DOC)
