# Supplementary material for: Pertussis Circulation Has Increased T-Cell Immunity during Childhood More than a Second Acellular Booster Vaccination in Dutch Children 9 Years of Age
Source: PLoS One. 2012 Jul 31;7(7):e41928. doi: 10.1371/journal.pone.0041928 (PMC3409203; doi:10.1371/journal.pone.0041928)
Supplement: Protocol S1 — Study protocol. (DOC) [file pone.0041928.s001.doc]

**PROTOCOL TITLE**

**The longitudinal kinetics of long term cellular memory immunity against *Bordetella pertussis* in Dutch 8-9 years old children after ACV booster vaccination.**

| **Protocol ID** | **Protocol LIS142** |
| --- | --- |
| **Short title** | **Cellular memory immunity DTP-IPV** |
| **Version** | **06** |
| **Date** | **28-01-2010** |
| **Coordinating investigators/project leader** | **Dr. A.M. Buisman (CIB, RIVM)**  **Investigators:**  **Dr. G.A.M. Berbers (CIB, RIVM)**  **Drs. L.H. Hendrikx (CIB, RIVM and Spaarne Ziekenhuis, Hoofddorp)** |
| **Principal investigator(s) (in Dutch: hoofdonderzoeker/uitvoerder)** | **Dr. M.A. van Houten**  **Kinderarts Spaarne Ziekenhuis Hoofddorp** |
|  |  |
| **Sponsor (in Dutch: verrichter/opdrachtgever)** | **National Institute of Health and the Environment (RIVM)** |
|  |  |
| **Independent physician(s)** | **Dr. T. Wolfs**  **Kinderarts WKZ/UMC Utrecht** |
|  |  |
|  |  |
| **Laboratory sites** | **1.RIVM**  **Center for Infectious Diseases (CIB)** |
|  |  |
|  |  |
| **Pharmacy** | **GlaxoSmitKline (GSK)** |
|  |  |

**PROTOCOL SIGNATURE SHEET**

| **Name** | **Signature** | **Date** |
| --- | --- | --- |
| **Sponsor or legal representative:**  ***National Instute for Public health and the Environment,***  ***Center for Infectious Disease (CIB),***  ***laboratory for Vaccine-preventable Diseases***  **For non-commercial research,**  **Head of Department:**  ***Dr . G. Loeber*** |  |  |
| **Coordinating Investigator:**  ***Dr. A.M. Buisman*** |  |  |
| **Principal investigator(s) (in Dutch: hoofdonderzoeker/uitvoerder)**  ***Dr. M.A. van Houten*** |  |  |

**TABLE OF CONTENTS**

1. INTRODUCTION AND RATIONALE [8](#__RefHeading___Toc94436779)

2. OBJECTIVES [11](#__RefHeading___Toc94436780)

3. STUDY DESIGN 12

4. STUDY POPULATION [13](#__RefHeading___Toc94436782)

4.1 Population base [13](#__RefHeading___Toc94436783)

4.2 Inclusion criteria [13](#__RefHeading___Toc94436784)

4.3 Exclusion criteria [13](#__RefHeading___Toc94436785)

4.4 Sample size calculation [14](#__RefHeading___Toc94436786)

5. TREATMENT OF SUBJECTS [15](#__RefHeading___Toc94436787)

5.1 Investigational product/treatment [15](#__RefHeading___Toc94436788)

5.2 Use of co-intervention (not applicable) [15](#__RefHeading___Toc94436789)

5.3 Escape medication (not applicable) [15](#__RefHeading___Toc94436790)

6. INVESTIGATIONAL MEDICINAL PRODUCT [16](#__RefHeading___Toc94436791)

7. METHODS [16](#__RefHeading___Toc94436800)

7.1 Study parameters/endpoints [16](#__RefHeading___Toc94436801)

7.1.1 Main study parameter/endpoint [16](#__RefHeading___Toc94436802)

7.1.2 Secondary study parameter *(vragenlijst)*

7.1.3 Other study parameters

7.2 Randomisation, blinding and treatment allocation [17](#__RefHeading___Toc94436805)

7.3 Study procedures [17](#__RefHeading___Toc94436806)

7.4 Withdrawal of individual subjects [18](#__RefHeading___Toc94436807)

7.4.1 Specific criteria for withdrawal *(not applicable)* [18](#__RefHeading___Toc94436808)

7.5 Replacement of individual subjects after withdrawal *(not applicable)* [18](#__RefHeading___Toc94436809)

7.6 Follow-up of subjects withdrawn from treatment *(not applicable)* [19](#__RefHeading___Toc94436810)

7.7 Premature termination of the study *(not applicable)* [19](#__RefHeading___Toc94436811)

8. SAFETY REPORTING [19](#__RefHeading___Toc94436812)

8.1 Vaccine information………………………………………………………………………. 16

8.2 Safety monitoring

9. STATISTICAL ANALYSIS [22](#__RefHeading___Toc94436819)

9.1 Descriptive statistics [22](#__RefHeading___Toc94436820)

9.2 Univariate analysis [23](#__RefHeading___Toc94436821)

9.3 Multivariate analysis [23](#__RefHeading___Toc94436822)

9.4 Interim analysis (if applicable)

10. ETHICAL CONSIDERATIONS [23](#__RefHeading___Toc94436824)

10.1 Regulation statement [23](#__RefHeading___Toc94436825)

10.2 Recruitment and consent [25](#__RefHeading___Toc94436826)

10.3 Objection by minors or incapacitated subjects (if applicable)……………………………. [25](#__RefHeading___Toc94436827)

10.4 Benefits and risks assessment, group relatedness [25](#__RefHeading___Toc94436828)

10.5 Compensation for Injury [25](#__RefHeading___Toc94436829)

10.6 Incentives (if applicable) [26](#__RefHeading___Toc94436830)

11. ADMINISTRATIVE ASPECTS AND PUBLICATION [27](#__RefHeading___Toc94436831)

11.1 Handling and storage of data and documents [27](#__RefHeading___Toc94436832)

11.2 Amendments [28](#__RefHeading___Toc94436833)

11.3 Annual progress report [28](#__RefHeading___Toc94436834)

11.4 End of study report [28](#__RefHeading___Toc94436835)

11.5 Publication policy [29](#__RefHeading___Toc94436836)

12. REFERENCES [30](#__RefHeading___Toc94436837)

**LIST OF ABBREVIATIONS AND RELEVANT DEFINITIONS**

| **ABR** | **ABR form (General Assessment and Registration form) is the application form that is required for submission to the accredited Ethics Committee (ABR = Algemene Beoordeling en Registratie)** |
| --- | --- |
| **AE** | **Adverse Event** |
| **AR** | **Adverse Reaction** |
| **CA** | **Competent Authority** |
| **CCMO** | **Central Committee on Research Involving Human Subjects** |
| **CV** | **Curriculum Vitae** |
| **DSMB** | **Data Safety Monitoring Board** |
| **EU** | **European Union** |
| **EudraCT** | **European drug regulatory affairs Clinical Trials GCP Good Clinical Practice** |
| **IB** | **Investigator’s Brochure** |
| **IC** | **Informed Consent** |
| **IMP** | **Investigational Medicinal Product** |
| **IMPD** | **Investigational Medicinal Product Dossier** |
| **METC** | **Medical research ethics committee (MREC); in Dutch: medisch ethische toetsing commissie (METC)** |
| **(S)AE** | **Serious Adverse Event** |
| **SPC** | **Summary of Product Characteristics (in Dutch: officiële productinfomatie IB1-tekst)** |
| **Sponsor** | **The sponsor is the party that commissions the organisation or performance of the research, for example a pharmaceutical**  **company, academic hospital, scientific organisation or investigator. A party that provides funding for a study but does not commission it is not regarded as the sponsor, but referred to as a subsidising party.** |
| **SUSAR** | **Suspected Unexpected Serious Adverse Reaction** |
| **Wbp** | **Personal Data Protection Act (in Dutch: Wet Bescherming Persoonsgevens)** |
| **WMO** | **Medical Research Involving Human Subjects Act (Wet Medisch-wetenschappelijk Onderzoek met Mensen** |

**SUMMARY**

**Rationale:** Since the incidence of whooping cough (pertussis) is increasing in the Netherlands, the effect of vaccination against *Bordetella pertussis* needs to be addressed.

Because of the increasing incidence of whooping cough at the age of 4, an acellular booster vaccination (ACV) at 4 years of age is introduced in the Netherlands in 2001. However, nowadays the peak incidence of whooping cough in children has shifted to 8-9 years old children. In addition, we also see a rise in notifications in adolescents and adults. Therefore, in some countries, e.g. Germany and France, an extra acellular booster vaccination has been given to the 9-14 years old children. Also in Belgium they will introduce an extra booster vaccination in 14-16 years old children. Because of the shift in the prevalence peak, the effect of the booster vaccination on the long term immunity against *Bordetella pertussis* needs to be addressed in this specific age group.

This study aims to investigate the longitudinal kinetics of the effect of the ACV booster vaccination on the memory B- and T- cell immunity in children who are primary vaccinated with whole cell vaccine (WCV) and boostered with ACV at 4 years old. Furthermore, the relationship between the cellular immunity and the antibody responses after ACV booster will be addressed in order to gain insight if further booster vaccinations are required.

**Objective:** The assessment of the duration of the cellular immunity to *Bordetella pertussis* after an extra ACV booster and the relationship between the memory B- cells and antibody responses.

The methods used in the study are:

B- and T- cell memory responses and antibody levels against the various components present in the acellular pertussis vaccine.

**Study design:** Intervention study: An extra ACV booster vaccination will be given to children of 8-9 years old. One pre-vaccination blood sample will be taken and two post-vaccination blood samples will be taken at 28 days and 1 year in which the frequency of memory B- cells will be assessed. Questionnaires including questions concerning clinical manifestations of whooping cough will also be completed. A control group including children of the same age who received the ‘normal’ DTP but did not receive the extra ACV booster at age 8-9 years will be included. One blood sample will be taken one year after the ‘regular’ DTP vaccination.

**Study population:**  A population of 8-9 years old children who received four vaccinations at 2,3,4 and 11 months with DT**Pwcv**-IPV-(Hib) and a booster vaccination at 4 years old with a three component ACV will be recruited.

**Intervention:** The combination vaccine DTPacv-IPV (Boostrix polio^TM^) produced by GSK containing a 3 component ACV (Pertussis toxin (Ptx), filamentous hemagglutinin (FHA) and pertactin (Prn), tetanus toxoid, diphtheria toxoid and inactivated polio virus, will be given 8-9 years old children who received the DT**Pwcv**-IPV-(Hib) at 2,3,4 and 11 months old and DTP + a three component ACV (Monovalent ACV by GlaxoSmithKline (GSK)) as a booster vaccination at 4 years old. The extra pertussis vaccination is combined with the DT-IPV and MMR vaccination which they receive in the regular immunization program. One pre- and two post-vaccination (28 days and 1 year) blood samples will be taken. In the control group one bloodsample will be taken one year after the regular DTP-vaccination.

**Main study parameters/endpoints:** The main study parameters will be pertussis specific memory B- and T- cell responses as well as antibody levels and affinity against the various proteins of pertussis and the other components of the DT**Pacv**-IPV-Hib vaccine.

**Nature and extent of the burden and risks associated with participation, group relatedness:**

Memory B- and T- cell immunity and the relationship between memory B- cells and antibody responses after a booster with an acellular pertussis vaccine will be addressed. This requires a blood sample of 15 ml per child pre- and post-vaccination (total of three blood samples) and for the control group one blood sample of 15 ml. A volume of 15 ml is needed to be able to do all the memory B- cell tests against the five most important proteins of *Bordetella pertussis*. With less material, B- cell responses against just one or two proteins of pertussis will be measured. A questionnaire will be used to relate immune functions to clinical manifestations of whooping cough.

We expect 5% of all parents to consent in participation based on our experiences with a previous study. The total number needed will be 70 children. For the control group 20 children will be included. There will be no risk for the participants.

**1. INTRODUCTION AND RATIONALE**

Pertussis is a serious disease caused by the bacterium *Bordetella pertussis (B. pertussis)* and more rarely by *Bordetella parapertussis*. Once *B. pertussis* has entered the host, it uses his adhesins to attach to cilia in the respiratory mucosa. When immunological constraints are absent, the organism will proliferate and it will begin to spread downward in the respiratory tract. During this time toxins are released, causing a highly contagious and severe infection of the respiratory tract (Weiss et al, 1996).

The detection of specific serum antibodies is the most widely applied method to investigate immunity against vaccine-preventable diseases like pertussis, although the presence of antibodies does not always confer protection for infection or disease. However, in large efficacy trials in Sweden and Germany both groups of investigators observed that the presence of antibodies against certain virulence factors of *B.pertussis* (in particular Pertussis toxin, Pertactin and Fimbriae) could be related to protection against whooping cough (Cherry et al 1998, Hewlett et al 1998, Storsaeter et al 1998). Nevertheless, antibodies decline very rapidly after booster vaccination against *B.pertussis*. This indicates increasing evidence for a central role of cellular mediated immunity against *B.pertussis*, although little data on that issue are available.

Moreover, since the peak incidence of whooping cough has moved from the 3-4 years old to the 9 years old, due to the introduction of the acellular booster vaccination at 4 years old, and the incidence of whooping cough also rises in adolescents and adults, the longitudinal kinetics of the effect of the acellular booster vaccine on the long term immunity needs to be addressed.

The pertussis vaccines are administered in the first year of life when the immune system is not yet fully developed and the (long term) effects of this major change in the vaccination program are not known.

This study aims to investigate the effect of an extra ACV booster vaccination on the longitudinal kinetics of the cellular immunity and antibody response, and to investigate a possible relation between the cellular and humoral immunity, in Dutch 8-9 years old children who are primarily vaccinated with the whole cell pertussis vaccination and boostered with the acellular pertussis vaccine at 4 years of age.

## Background

*B. pertussis* occupies the respiratory tract in humans. Previous studies indicate that both antibody-mediated and cellular immune mechanisms play a large role in protection against pertussis (Leef et al, 2000). Humoral antibody responses are largely measured to the vaccine-components pertactin (Prn), pertussis toxoid (Ptx), Filamentous hemaglutin (FHA) and fimbriae (Fim). These antigens neutralize, either have toxic effects or inhibit attachment of the bacterium to the epithelial cells.

The intracellular positioning of *B. pertussis* requires elimination of the cells that are infected; an activity, mainly associated with cellular immunity (Mills et al, 2001, Zepp et al, 1997).

Immunization using a vaccine composed of whole cells of killed *Bordetella pertussis* (WCV) bacteria was introduced in the National Immunization Programme (RVP) in 1957. In 1962 the combination vaccine DPwcvT-IPV was introduced. Since 1997 many changes have occurred in the pertussis vaccination in the National Immunization Program, which are listed below.

Changes in pertussis vaccination programme in the Netherlands since 1996

end of 1997: * 3,4,5 and 11 months: D**Pwcv**T-IPV + Hib potency 4→ 7 IU

1999: * 2,3,4 and 11 months: D**Pwcv**T-IPV + Hib adaptation schedule

2001: * 2,3,4 and 11 months: D**Pwcv**T-IPV + Hib

* 4 years booster: DT-IPV + **Pacv** Monovalent ACV (GSK)

2003: * 2,3,4 and 11 months: D**Pwcv**T-IPV-Hib combination

* 4 years booster: DT-IPV + **Pacv** Monovalent ACV (GSK)

2005: * 2,3,4 and 11 months: D**Pacv**T-IPV-Hib Infanrix-IPV-Hib (GSK)

HepB (SP)

* 4 jaar booster: DTP + **Pacv** Monovalent ACV (GSK)

2006: * 2,3,4 and 11 months: D**Pacv**T-IPV-Hib Pediacel (SP)

* 4 years booster: D**Pacv**T-IPV Triaxis (SP)

Infanrix Hexa (HepB)

2007: * 2,3,4 and 11 months: D**Pacv**T-IPV-Hib Pediacel (SP)

* 4 years booster: D**Pacv**T-IPV Triaxis (SP) /

Infanrix-IPV (GSK)

Nowadays, children are vaccinated at the age of 2, 3, 4, and 11 months with a tetravalent diphtheria, pertussis, tetanus and inactivated polio virus vaccine which is combined with a Hib vaccine (DTP-IPV-Hib) prior to vaccination (Labadie et al 1996). This mass vaccination has markedly decreased the incidence of pertussis and the mortality caused by the disease.

In the Netherlands, a rise in notifications of whooping cough is observed every 2-3 years since 1996 (de Greeff et al, 2004). As a response to the first epidemic of 1996-1997 two changes have been made in the RVP. Firstly, in 1997 the potency of the whole cell pertussis vaccine component was enhanced from 4 to 7 International Units and the production process has been changed in order to enlarge the Ptx content of the vaccine. Secondly, in 1999 the immunization schedule was shifted forward from 3, 4, 5 months to 2, 3, 4 months to provide protection for the neonates as early as possible.

Adverse reactions observed with WCV were a great stimulus for the development of ACVs, which reduced the severity and number of adverse reactions. In addition, as serological studies showed the presence of very low titers against pertussis antigens in children at the age of 4 years (Berbers et al, 1999), in 2001 it was decided to introduce an acellular booster at 4 years of age.

Since January 2005, because of the public commotion about the adverse reactions to WCV, all children receive DTPacv IPV-Hib vaccine (Infanrix-IPV) from GSK containing a 3 component ACV (Ptx, FHA and Prn) at 2,3,4 and 11 months of age. Since January 2006, the combination vaccine DTPacv-IPV-Hib (Pentacel) of Sanofi Pasteur containing a 5 component ACV (Ptx, FHA, Prn and Fim2 + 3) is used.

However, although antibody titers are higher after vaccination with ACV, they wane relatively fast to undetectable levels (Berbers et al, 1999) which indicates that cellular immunity plays an important role in maintaining long term immunity. Our ongoing study (Memory study, protocol number LTR 137) shows high antibody titers against the 5 pertussis antigens 1 month after acellular booster vaccination at 4 years of age. However, antibodies decrease 2 years after the acellular booster and more than 90% are below protection level at 9 years of age (Hendrikx et al, in progress).

Furthermore, when administering ACV, it appears that the type of immune response induced differs from children whom are vaccinated with WCV (Olin et al, 1997; Simondon et al, 1997).

Nowadays, the peak prevalence of whooping cough is noticed in 9 year old children and young adolescents, due to the booster at 4 years of age (De Greef et al, 2008). Therefore, in Germany and Sweden an extra ACV booster vaccination is given at 9-10 years of age ([www.euvac.net](http://www.euvac.net/)) and in France and Italy at 10-11 years of age. In Belgium they also will introduce an acellular booster vaccination at 14-16 years of age. The effect of an extra ACV at this age on the (long term) immunity is still unknown.

The immune system is still in development when the first vaccinations take place.

Vaccination against disease often aims for the production of high antibody titers, and adjuvants are regularly included for their capacity to induce antibody-mediated protection. This may however not be the most efficient immune response elicited. Evidence is accumulating that it is very important to elicit the right type of immune response when vaccinating individuals in order to be optimally protected. When aiming towards high antibody production it is important to realize to what extent antibodies mediate protection and the time period they remain protective. Growing insights in the immune system have revealed that the innate immune response has a regulating role in induction of memory B- cells as well as the different types of T- lymphocytes, which could have important implications for the development and production of a vaccine.

# This project aims to get insight in the long-term protective memory immunity against *B. pertussis* and the relationship between the cellular and antibody immune responses in children who completed the vaccination program and who will receive an extra booster with ACV. Therefore we will investigate the longitudinal kinetics of the effect of the ACV booster on the cellular memory immunity as well as the relation with the antibody response.

# 2. OBJECTIVES

Primary Objective:

To assess the longitudinal kinetics of the long term cellular immunity to *B. pertussis* after ACV and the relationship between the cellular (long term) immunity and antibody responses.

There is little knowledge of the cellular immune response against *B.pertussis* in Dutch children. These data are very relevant to obtain insight in the effect of vaccination on the long term protection against *B. pertussis* as well as the effect of changes in the vaccination program on the immune status of children.

The primary objective of this study is to get insight in the longitudinal kinetics of the B- and T- cellular memory immune responses against *B.pertussis* and the relationship with the antibody response induced after an extra booster vaccination with ACV at 8-9 years of age.

- Memory B- and T- cells will be isolated and after polyclonal stimulation will be tested against various proteins of B. pertussis.
- Antibody responses will be measured against the various proteins of B.pertussis.

Secondary Objective:

If there are enough lymphocytes, the immune response (memory B- and T-cells and antibody responses) to other vaccine preventable diseases, like measles, mumps, diphtheria, tetanus and polio will also be measured.

**3. Study design**

Interventional study with a single intervention (an extra DTPacv-IPV booster vaccination in the 8-9 years old children) and blood samples taken pre-vaccination and post-vaccination at 28 days and 1 year after the extra booster. The time window for the blood sample at 28 days will be ± 2 days and the time window for the blood sample 1 year after vaccination will be ± 2 weeks. A questionnaire will be taken about the clinical symptoms of whooping cough (annex 4).

A control group including children of the same age who did not receive the extra ACV booster at age 8-9 years will be included. One bloodsample will be taken one year after the ‘regular’ DTP vaccination.

# 4. STUDY POPULATION

## Population (base)

A population of healthy Dutch 8-9 years old children will be recruited. The children have already received four vaccinations at 2, 3, 4 and 11 months with DTPwcv-IPV-Hib and a booster vaccination with a three component ACV at 4 years.

## Inclusion criteria

Infants in good general health (eligible) who have been vaccinated according to the Dutch national immunization program.

Provision of written informed consent by both parents and legal representatives.

## 4.3 Exclusion criteria

## Any of the following criteria will exclude a volunteer from participation, at start of the study:

- Present evidence of serious disease(s) demanding immunosuppressive medical treatment, like corticosteroids that might interfere with the results of the study within 3 months.
- Any known primary or secondary immunodeficiency.
- Boostrix Polio must not be given to people with a known hypersensitivity after a former injection of diphteria-, tetanus-, pertussis- or poliomyelitis-vaccins or one of the substances of the vaccines.
- Boostrix Polio is contraindicated to people who suffered from an encephalopathy without a known cause within 7 days after a former pertussis vaccination.
- Boostrix Polio must not be administered to people who suffered from a temporary trombocytopathia or people who had neurologic complications (convulsions or hypotonehyporesponsive episodes) after a former administration with a diphtheria or tetanus vaccine.

Delay criteria

In case of a child is having fever (> 38,5^o^C) within 2 days before the vaccination will take place and before blood sampling, which can interfere with the cellular immune responses at that time, another appointment for the vaccination and/or blood sampling will be made. If the child has received another vaccine within a month before the vaccination and/or the blood sample will be taken, the vaccination and/or blood sample will be delayed too.

## Sample size calculation

This study is designed to investigate the direct as well as the long term effect of the acellular pertussis vaccine on the cellular immunity by measuring the cellular immune responses and the antibody titers after primary series and an extra booster vaccination.

Data on cellular immune responses against *B. pertussis* in Dutch children are not yet available.

An ongoing study that compares the effect of WCV and ACV on the long term cellular memory immunity against *B. pertussis* and the other components of the DTP-IPV-Hib vaccine in Dutch children, started in 2006 and aims for 60 children per age group. To compare these data with the data that will be attained in this study, we aim for a total of 60 children. However, we expect about 15% of the children to stop prematurely; therefore we aim for a total of 70 children.

For a control group we aim for 20 children. A group of 20 children of 10 years of age not being boostered with a pertussis vaccine at 9 years of age will be included in the study. If circulation of pertussis will be increased during the last year, the geomean antibody titers against pertussis toxin (PT) will be increased in our control group in 2010 compared to 9 years old children before booster vaccination in 2009. Moreover, in addition to PT antibody titers the percentage of PT specific memory B-cell responses will increase too due to a higher circulation of pertussis (Buisman et al, Vaccine 2009). However, a high increase in the circulation of pertussis is very unlikely.

Our previous studies in 2007 showed equal geometric mean antibody titres in children of 6 to 10 years of age, in groups of 60 children as well as in groups of 20 children. Therefore, the number of 20 children in this control group will be sufficient to calculate any effect of recent circulation of pertussis on pertussi toxin specific antibody titers or memory B-cell responses.

# 5 TREATMENT OF SUBJECTS

## 5.1 Investigational treatment

#### Children of 8-9 years old will be asked to receive an extra pertussis-component in the DT-IPV booster vaccination (DT*Pacv*-IPV) together with their MMR vaccination. There will be no extra injection, only a change in vaccination composition (addition of the pertussis component). Pre- and post-vaccination a blood sample of 15 ml will be taken by venous puncture. Post-vaccination blood sampling will be done at 28 days (± 2 days) and 1 year (± 2 weeks) after the booster vaccination. To examine *B. pertussis* specific memory B- and T-cells we need a minimum of 1 x 10^6^ Peripheral Blood Mononuclear Cells (PBMC's) to analyse one of the 5 most important *B. pertussis* specific antigens (Ptx, Prn, FHA, Fim 2 and 3). B- cells account for only 2 to 10% of all PBMC's and only 20-30% of all B- cells are memory B- cells. On average we can isolate 1 x 10^6^ PBMC's per ml blood, however this is highly variable per person. To analyse specific memory B- cells to all 5 *B. pertussis* antigens, an amount of 15 ml blood is needed. With less material, B- cell responses against just one or two proteins of *B. pertussis* (Ptx, Prn or FHA) will be measured. In our ongoing study a blood sample of 15 ml is not a problem in 8-9 years old children.

##

## 5.2 Use of co-intervention (if applicable)

An extra ACV booster vaccination including a 3 components ACV combined by GSK (DTPacv-IPV, Boostrix^TM^) will be given together with their MMR vaccination that they receive according to the RVP.

**5.3 Escape medication (if applicable)**

Optionally, a local anaesthetic (Emla® crème; AstraZeneca) will be used to minimize the pain of the venous puncture.

# INVESTIGATIONAL MEDICINAL PRODUCT

- Product: Boostrix Polio

Boostrix Polio is indicated for booster immunization against pertussis, diphteria, tetanus and polio as a single dose in individuals 4 years of age and older.

Each dose of 0,5 ml Boostrix Polio for intramuscular injection contains 2 IE Diphteria Toxin (Dtx), 20 IE Tetanus toxin (Ttx), 8 µg Ptx, 8 µg FHA, 2,5 µg Prn, 40 D-antigenunits IPV-1, 8 D-antigenunits IPV-2 and 32 D-antigenunits IPV-3 adsorbed onto 0,3 mg aluminimumphosphate.

### - How supplied Boostrix Polio is supplied in a glass vial with an elastic cap as provided by GSK.

# - Dosage and administration

Boostrix Polio must be at room temperature before administration. Before administration, shake vigorously to obtain a homogeneous, turbis, white suspension. Do not use if resuspension does not occur with vigorous shaking. Before injection, the skin at the injection site should be cleaned and prepared with suitable germicide.

Boostrix Polio should be administered as a single 0,5 ml injection by intramuscular route into the deltoid muscle of the upper arm.

- Storage

Boostrix Polio will be provided by GSK.

Boostrix Polio must be stored refrigerated between 2°C and 8°C. Boostrix Polio must not freeze and must be discarded if the vaccine has been frozen. Boostrix Polio must not be used after expiration date shown on the label. Damaged vials must be returned to GSK.

# METHODS

## 7.1 Study parameters/endpoints

#### 7.1.1 Main study parameter/endpoint

The main study parameters will be the longitudinal kinetics of B- and T- cell memory immune responses and antibody responses against the vaccine proteins of *B. pertussis*.

Blood samples will be separated in Peripheral Blood Mononuclear Cells (PBMC's) and plasma samples. PBMC's will be divided in purified B- cell populations and T- cell populations.

B- cells will be cultured and memory B- cells will be polyclonal stimulated (Bernasconi et al, 2002). After 5 days stimulation, B- cell memory responses will be measured against the various proteins of *B. pertussis* (FHA, pertactin, PT, Fim 2 and 3) by ELIspot assays and MIA (Multiplex Immuno Assay) or ELISA of the culture supernatants.

The plasma samples will be used to measure antibody responses against the various proteins of

*B. pertussis* (PT, PRN, FHA, Fim 2 and 3), and, if necessary to the extra components of the National Immunization Program. IgG and IgA antibody titer is measured in

multiplex immuno assay (MIA) (Gageldonk et al 2008) in two dilutions using FDA reference

serum as standard (EU/ml, Meade et al 1995).

## Randomisation, blinding and treatment allocation

No randomization or different treatment groups are designed in this study.

Personal data

Personal (identity) data are defined as the set of data that together might disclose the identity of a participant: name, date of birth, sex, and full address. The principal clinical investigator will guarantee that personal data from participants are treated anonymously, unless medical conditions of the participants require release of anonymity.

Study data

Study data are defined as the set of all participant-related variables such as: initials, postal code (only the four numbers), vaccination status, data on possible adverse events, antibody data. The storage of data is such that third parties have no access to the data.

- Data on the CRF

On the CRF the following data must be written:

- UTN (unique trial number)

- number of vial

- expiration date

- place of administration (left or right arm)

- name, date, signature of the person who have given the vaccination.

- remarks

## Study procedures

Since the most cases of *B. pertussis* infection occurs in 9 years old children nowadays, it is important to monitor the immune system of children in this age group. Study procedures will be done by qualified persons according to standard operating procedures.

The booster vaccination and blood samplings using venous puncture (15 ml) are performed by experienced physicians and study nurses. A local anesthetic (Emla crème) will be used to minimize the pain of the venous puncture. In case of resistance the behavior code of the Dutch Society of Pediatricians will be followed.

Parents will be asked to participate in the study by letter. When they fill in a reply-card, they will receive an informed consent by mail and will subsequently be contacted to make an appointment for their first visit in the pediatric outpatient’s clinic. During this visit, both parents will be asked to sign an informed consent after which the blood sampling will be done and the vaccination will be given. During the next visits at 28 days (± 2 days) and 1 year (± 2 weeks) after the vaccination, a venous blood sampling will be done. When the blood sampling will be difficult, the sampling will be stopped immediately.

Because we only need 20 children in the control group, we will recruit these children from the group of children who were not able to participate at the start of the Booster study, because we had too many registrations. We will contact the parents of these children again by phone. In addition we will send a newsletter (annex 1) to each participant of the Booster study to inform about the progress of the study and in which we also call up for children who could participate in the control group of the Booster study. When parents are interested in participation, we will sent the extra information (annex 2) about the study and the control group and we will make an appointment for blood sampling in the Spaarne hospital. During this visit, both parents will be asked to sign an informed consent for the control group (annex 3) after which blood sampling will be done. Data will be filled in the CRF for the control group (annex 4).

## Withdrawal of individual subjects

Parents of subjects can decide not to participate at any time for any reason without any consequences. The investigator can decide to withdraw a subject from the study for urgent medical reasons.

### Specific criteria for withdrawal (if applicable) not applicable

## Replacement of individual subjects after withdrawal

## Because we expect 15% of all children to prematurely withdraw from the study, we aim for 70 children at the start of the study, so there will be 60 children completing the study. For the control group we aim for 20 children.

## Follow-up of subjects withdrawn from treatment no follow-up

## Premature termination of the study

Because Boostrix-polio is a registered vaccine which is already used in other countries in the same age group (children 9-12 years of age), we don’t expect that serious side effects will occur. Therefore we don’t expect to premature terminate the study.

**8 SAFETY REPORTING**

**8.1 Vaccine information**

8.1 Study Product Accountability

Each time vaccines are used, a signed form with information on the quantity, expiration date and vial numbers of the delivered vaccines must be added. The form must be dated en signed by the person who is responsible for storage and the person who receives the product. The person who gives the vaccine is responsible for the accountability at the location.

If at the end of the study a discrepancy occurs between the number of delivered vaccines and the number of vaccine used, a written explanation must be obtained.

All unused vaccines will be returned to GSK at the end of the study.

8.2 Safety monitoring

# - Safety monitoring

Each child will be observed during 15 minutes after vaccination by the study nurse or doctor who gave the vaccination.

- Adverse events

Any untoward medical occurrence in a patient or clinical investigation subject administered a pharmaceutical product and which does not necessarily have a causal relationship with this treatment. An adverse event (AE) can therefore be any unfavourable and unintended sign (including an abnormal laboratory finding), symptom, or disease temporally associated with the use of a medicinal (investigational) product, whether or not related to the medicinal (investigational) product (see the ICH Guideline for Clinical Safety Data Management: Definitions and Standards for Expedited Reporting).

An serious adverse event is defined as each unexpected change in the medical situation, which

- causes death
- is live threatening
- causes admission to a hospital or which delays a current admission
- results in ongoing negative causes or handicaps

This classification will be used for the relation with the given vaccine

- Unlikely. There is no medical evidence suggesting the adverse event is related to the vaccination or another, more plausible explanation.
- Possible. There could be a possible medical explanation that the event is related to the vaccination. However, other medical explanations can not be excluded.
- Likely. There is a potent medical explanation for a causal relation between the administration of the vaccination and the adverse event. There are no possible other explanations for the adverse event.

Each SAE that occurs within a month after administration of the vaccine will be reported at the research centre and carefully registered and examined by the research group.

In case of a Suspected Unexpected Serious Adverse Reaction (SUSAR) that might be related to the administration of the vaccine, will be immediately reported to the vaccine distributor GSK and within 14 days to the CBG with a CIOMS form. The SUSAR will also be reported to the CCMO and the BI (Bevoegde Instantie).

The research doctors (M.A. van Houten and L.H. Hendrikx) can evaluate whether a SAE is a SUSAR.

- Adverse reactions Boostrix Polio

In clinical trials in which Boostrix Polio had been administered to adolescents and adults, most common adverse events within 24 hours after administering are:

very often >10%: pain, erythema and swollen site of the injection, fever (T>37,5°C), fatigue

often 1-10%: fever (T>39,0°C) in 4-8 year old children, erythema and swollen site of the injection,

malaise

# - Documentation SAE and SUSAR

All adverse events or complications as been noticed by the parents, caregivers or research nurse or doctor must be written in the CRF.

Serious adverse events need to be reported on the SAE form as well.

The following criteria the adverse events will be used:

| Local reactions | Intensity | size of local reaction |
| --- | --- | --- |
| Redness and swollen | Mild | < 10 mm |
|  | Moderate | 10-29 mm |
|  | Severe | > 30 mm |

| Systemic reactions |  |  |
| --- | --- | --- |
| fever | 1 | 37,5-38,4 °C |
|  | 2 | 38,5-39,4 °C |
|  | 3 | 39,5-40,4 °C |
|  | 4 | 40,5 °C |
| Persistent, prolonged crying | Mild | 1 hour |
|  | Moderate | 1-3 hours |
|  | Severe | 3 hours |
| hyporesponsive episode | Severe | 3 hours |
| less active | yes/no |  |
| recuced apitite | yes/no |  |
| vomiting | yes/no |  |
| Diarhoea | yes/no |  |

**9.** **STATISTICAL ANALYSIS**

## Descriptive statistics

Since nothing is known about cellular immune responses against *B. pertussis* in the Dutch population, data of these responses after the booster vaccination with DTPacv-IPV at 8-9 years as well as in the control group will be presented in descriptive statistics, including frequency tables and charts. Descriptive statistics will mainly constitute measures of central tendency (e.g. mean) and measures of dispersion (e.g. range and standard deviation). Antibody levels against the different pertussis antigens will be described in figures and geometric means will be calculated. Differences between the antibody levels pre- and post vaccination will be analysed with a paired t-test. The cellular responses will also be described in figures. Correlations will be measured between the cellular responses and the antibody levels.

Considering the number of children who are already included in the previous study (60 participants) and the expectation of 15% of the children will quit the study prematurely, we aim for 70 children.

The number of 60 children per age group in the former Memory study, is calculated as follows:

To be able to describe significant differences in serological responses between groups or time-points per group, the size of the study groups is dependent on the number of data available from former studies with the “old” WCV and the observed mean antibody titers against the pertussis vaccine components. Data from the DTPwcv/Hib “mixture” study performed in 1993-1994 (Labadie et al 1996) are available from 90 samples. If we combine these data with results from MMR studies (also pertussis measured) a total of 268 data are available. Inclusion of 45 children in each group in this study would mean that there has to be a 2.3-fold rise in mean antibody titers for antibodies against PT and a 2.0-fold rise in that for PRN between groups (power of 90% and a significant level of 5%) using data from the DTwcP/Hib mixture study only (n=90). If also the other available data (n=268) are included these titer rises would become 2.1-fold for PT and 1.8-fold for PRN needed to detect a significant difference with the same power and level. Recent experiments has clearly indicated that at 28 days after vaccination significant differences in antibody responses were found between groups consisting of 35 children of 4 years, which had received either Triaxis or Infanrix-pertussis as a booster vaccine.

Considering the available time span and the area’s where the study has to be performed it is realistic to aim for 45 children per group. Anticipating withdrawals and dropouts during the trial the objective is to include 60 children per group. This will allow us to observe a statistically significant difference between the different time-points before and after booster vaccination if we measure a twofold rise in the antibody titer against these antigens of the pertussis vaccine component.

For the control group we aim for 20 children who did not receive the extra ACV booster at age 8-9 years and who have the same age as the children under investigation in the study. One bloodsample will be taken one year after the regular DTP vaccination to exclude the effect of *Bordetella pertussis* circulation on the immunological parameters measured. Since pertussis antibody levels wane in 2-3 years after vaccination, high antibody levels will indicate a recent infection with *Bordetella pertussis* in this control group. For comparison of the results of the children who received the extra pertussis vaccination with this control group one-year post-vaccination, we will use an unpaired t-test. Antibody levels and cellular responses will be described in descriptive statistics and figures. In this way, we can improve the understanding of the vaccine effect on the immunological parameters measured.

## Univariate analysis

The significance of correlations will be analysed by the nonparametric Spearman test.

## Multivariate analysis

The data from the questionnaires will be related to the cellular and serological responses measured.

# ETHICAL CONSIDERATIONS

## Regulation statement

This clinical study will be performed according to the current rules for Good Clinical Practice (GCP), as described by the Committee for Proprietory Medical Products (CPMP) of the European Union and the International Committee on Harmonisation (ICH) in "Note for Guidance on Good Clinical Practice, document CPMP/ICH/135/95”, effective since January 17th 1997 and according to the Dutch Medical Research Involving Human Subjects Act (WMO), under the general ruling of the Clinical Trial Directive of the EU (2001/20/EU). These rules include the ethical guidelines described in the "Declaration of Helsinki" (World Medical Association Declaration of Helsinki, Recommendations guiding physicians in biomedical research involving human subjects. Adopted by the 18th World Medical Assembly (WMA), Helsinki, Finland, 1964; amended by the 29th WMA, Tokyo, Japan, 1975; 35th WMA, Venice, Italy, 1983; 41st WMA, Hong Kong, 1989, the 48th General Assembly, Sommerset West, Republic of South Africa, 1996 and the 52nd General Assembly, Edinburgh, Scotland, 2000).

The principal clinical investigator will maintain the privacy of the participant and will be responsible for the welfare of the participant. An experienced physician together with a nurse will perform the vaccination and the blood samplings.

The study proposal will be submitted to the CCMO for review and approval of the clinical investigation and the methods and materials used to gather and register the declaration of informed consent of the participants. Both parents have to sign the informed consent to let their child participate in the study. The study will not start before written approval of the CCMO has been obtained.

Protocol modifications in the ongoing study which affect the safety of the participant, or which alter the scope of the investigation, the scientific quality of the study, the experimental design, assessment variables, the number of participants or the selection criteria, will be made only after appropriate consultation with the Clinical Trial Monitor, the Sponsor/investigator, and the Principal Clinical Investigator.

The CCMO and the BI will be informed of any protocol modification and of severe or unexpected unwanted events that occur during the study and that may adversely affect the safety of the participants or which alter the scope of the investigation, the scientific quality of the study, the experimental design, the number of participants or the selection criteria and the performance of the investigation.

Changes in the protocol will only be made by written amendment agreed upon between the Clinical Trial Monitor, the Sponsor/investigator, and the Principal Clinical Investigator. The amended protocol will not be implemented before approval of the CCMO.

It is the responsibility of the Principal Clinical Investigator to adequately explain the volunteer the aims, methods, anticipated benefits (for participants and/or others), potential discomforts of the study and treatments, both in words and by written information. They will also have the possibility to contact medical doctors for additional information (annexes 2 and 3).

Furthermore, the principal clinical investigator should specifically inform the participant:

- That the privacy of the participant will be maintained by coding the identity. The informed consent implies that participant grants leave to inspect the personal information registered during the study; with access explicitly only to members of the Clinical Research Unit, to the Clinical Trial Monitor, the Medical Ethics Committee and by a qualified authority in case of an audit. The registered information will be archived during a period of 15 years.
- That he/she is completely free to refuse to enter the study at any time, without giving a reason and without consequences for treatment in the event that problems arise.
- On procedures for compensation entitlement in the event of health damage or disablement due to study participation.
- About all relevant information available during the study.

## Recruitment and consent

The parents or legal representatives of 8-9 years old children who participate in the Dutch Vaccination Programme (RVP) will be informed about the study by mail (annex 1A). If they would like to participate in the study they will fill in a reply card. After receiving this card we will explain the parents about the study proposal schedule and (dis)advantages (patient information form (PIF), annex 1B) by telephone and mail. After at least one week a second telephone call will be made. If parents agree in participation, the in- and exclusion criteria will be checked and subsequently an appointment for the booster vaccination and blood sampling at the pediatric outpatient’s clinic in the Spaarne Hospital in Hoofddorp will be made. If the parents or legal representatives of the infant has given informed consent and meets all the criteria, he/she will enter the study as a participant, and will be given a Unique Trial Number (UTN). The case report form (CRF) (annex 2) contains a section for documenting the procedure of informed consent and must be completed by the investigator.

The principal clinical investigator will obtain signed informed consent from each volunteer that indicates that he/she is willing to participate in the study. The volunteers should have sufficient time to consider their decision or consult a third party. A copy of the text of the informed consent, in Dutch, will be submitted to the CCMO (annex 3).

During the visit the PIF will be reviewed and discussed with the investigator. After a written consent for participation is signed by both parents of the child, the blood sampling (15 ml) and the vaccination will be given. An appointment for the blood sample at 28 days (± 2 days) will be made.

During the second visit a blood sample of 15 ml will be taken. After 1 year the investigator will contact the parents again to make an appointment for the last visit at which another blood sample of 15 ml will be done.

The control group will be recruited from the children who were not able to participate at the start of the Booster study, because we had too many registrations. We will contact the parents of these children again by phone. In addition we will send a newsletter (annex 1) to each participant of the Booster study to inform about the progress of the study and in which we also call up for children who could participate in the control group of the Booster study.

## Objection by minors or incapacitated subjects (if applicable)

## Benefits and risks assessment, group relatedness

## Compensation for injury

According to a Ministerial Order, RIVM is excluded from compulsory insurance for clinical research as determined by the Dutch law on Medical Investigations (WMO, section 7, paragraph 6). Participants can recover the loss from RIVM. Any claims will be settled according to the terms of an insurance company. Participants will be informed about these terms in detail in the Participant Information Form.

The sponsor has a liability insurance which is in accordance with the legal requirements in the Netherlands (Article 7 WMO and the Measure regarding Compulsory Insurance for Clinical Research in Humans of 23rd June 2003). This insurance provides cover for damage to research subjects through injury or death caused by the study.

1. € 450.000,-- (i.e. four hundred and fifty thousand Euro) for death or injury for each subject who participates in the Research;
2. € 3.500.000,-- (i.e. three million five hundred thousand Euro) for death or injury for all subjects who participate in the Research;
3. € 5.000.000,-- (i.e. five million Euro) for the total damage incurred by the organisation for all damage disclosed by scientific research for the Sponsor as ‘verrichter’ in the meaning of said Act in each year of insurance coverage.

The insurance applies to the damage that becomes apparent during the study or within 4 years after the end of the study.

## Incentives (if applicable)

In public health view, participation in this study will contribute to the confidence in the Dutch Vaccination Programme. Children will receive a present of 10 euro after the first post-vaccination blood sample and 15 euro after the second post-vaccination blood sample for their participation.

Children who participate in the control group will receive 10 euro for the single blood sample.

# ADMINISTRATIVE ASPECTS AND PUBLICATION

# 11.1 Handling and storage of data and documents

All study data will be registered in a collection of files (the source documents). Separate records will be commenced for each participant. All clinical data on participants obtained from the handling, treatment and observation will be recorded on Case Report Forms (CRF). Personal identifiers will not be recorded on the CRF, with exception of date of birth, initials, postcode and gender. The Principal Clinical Investigator assures that the anonymity of the participants is maintained; keeping separate log of codes, names and addresses of participants. The CRF forms the basis for further analysis of the study results. The contents of the CRF are summarized in annex 2. To enable efficient data analysis, electronic data files will be compiled. Source documents and hard copies of electronic files/analyses will be stored according to GCP guidelines (for a period of 15 years).

The CRF will contain information obtained according to the study assessments described in Chapter 4. The sponsor investigator is not entitled to know personal data of the participant. Thus, the principal clinical investigator is required to separate personal and study data on the CRF. Throughout the CRF, on every page, the UTN number will be used as the unique participant identifier. Furthermore, date and time of study procedures and assessments will be recorded throughout the CRF for all recorded observations.

Data registered in the source documents will be made available for analysis in electronic data files. To establish a validated data set for analysis, a procedure of entry and verification will be used. All necessary changes and corrections after data entry will be motivated, dated and signed by the investigators.

Data analysis will be done by the sponsor investigators. Validated data sets will be used for analysis. All obtained data will be used for intention to treat analysis.

The study results will be reported in an internal report and submitted for publication in peer-reviewed journals. Publications will be drafted by the sponsor investigators.

## Amendments

A ‘substantial amendment’ is defined as an amendment to the terms of the METC application, or to the protocol or any other supporting documentation, that is likely to affect to a significant degree:

- the safety or physical or mental integrity of the subjects of the trial;
- the scientific value of the trial;
- the conduct or management of the trial; or
- the quality or safety of any intervention used in the trial.

All substantial amendments will be notified to the METC and to the competent authority.

Non-substantial amendments will not be notified to the accredited METC and the competent authority, but will be recorded and filed by the sponsor.

This is the first amendment of this study. The changes are:

- Change in our reference: LIS142 (instead of LIS/IMM138)

- The inclusion of a control group consistent of 20 children of the same age as the participants, but who received the regular DTP vaccination and did not receive the extra ACV booster vaccination at 8-9 years of age.
- The newsletter that we would like to sent to each participant of the Booster study (annex 1)
- The extra information for the control group (annex 2)
- The informed consent form for the control group (annex 3)
- The CRF for the control group (annex 4).
- The question list for the control group (annex 5)

## Annual progress report

The sponsor/investigator will submit a summary of the progress of the trial to the accredited METC once a year. Information will be provided on the date of inclusion of the first subject, numbers of subjects included and numbers of subjects that have completed the trial, serious adverse events/ serious adverse reactions, other problems, and amendments.

**11.4 End of study report**

The investigator [sponsor] will notify the accredited CCMO [and the competent authority] of the end of the study within a period of 8 weeks [90 days]. The end of the study is defined as the last patient’s last visit.

In case the study is ended prematurely, the investigator [sponsor] will notify the accredited CCMO [and the competent authority within 15 days], including the reasons for the premature termination.

Within one year after the end of the study, the investigator/sponsor will submit a final study report with the results of the study, including any publications/abstracts of the study, to the accredited CCMO [and the Competent Authority].

**11.5 Publication policy**

The coordinating investigators will submit the results of the study for publication in collaboration with the principal investigators

# REFERENCES

Ausiello CM, Fedele G, urbani F, Lande R, di Carlo B, Cassone A. native and genetically inactivated pertussis toxins induce human dendrtitic cell maturation and synergize with lipopolyssacharide in promoting T helper type 1 reponses. J. Infect Dis 2002; 186: 351-60

1. Ausiello CM, Lande R, la Sala A, Urbani F, Cassone A. Cell-mediated immune response of healthy adults to *Bordetella pertussis* vaccine antigens. J Infect Dis. 1998 Aug;178(2):466-70.
2. Berbers G.A.M., Lafeber A.B., Labadie J., Vermeer-de Bondt P.E., Bolscher D.J.A. & Plantinga A.D. A randomized controlled study with whole-cell or acellular pertussis vaccines in combination with regular DT-IPV vaccine and a new poliomyelitis (IPV-Vero) component in children 4 years of age in the Netherlands. RIVM report 105000.001 (1999).

Bernasconi NL, E. Traggiai, A. Lanzavecchia. Maintenance of serological memory by polyclonal activation of human memory B cells. Science 2002, 298:2199-2202.

1. Canthaboo C, Williams L, Xing DK, Corbel MJ. Investigation of cellular and humoral immune responses to whole cell and acellular pertussis vaccines. Vaccine. 2000 Nov 8;19(6):637-43.
2. Cherry, J. D., J. Gornbein, U. Heininger, and K. Stehr. A search for serologic correlates of immunity to *Bordetella pertussis* cough illness. 1998. Vaccine 16**:**1901–1906.

Dagan R,D Goldblatt, JR. Maleckar,Mansour Yaïch,and J Eskola. Reduction of Antibody Response to an 11-Valent Pneumococcal Vaccine Co administered with a Vaccine Containing Acellular Pertussis Components. Infect Immun 2004, 72, 8383-5391.

1. de Greeff SC, JFP Schellekens, FR Mooi and HE de Melker. Pertussis in The Netherlands, 2001-2003. 2004. RIVM Report 1285071010/2003
2. Gruber C, Lau S, Dannemann A, Sommerfeld C, Wahn, U, Aalberse RC. Down regulation of IgE and IgG4 antibodies to tetanus toxoid and diphteria toxoid by covacciantion with cellular *Bordetella pertussis* vaccine J. Immunol 2001;167:2411-
3. Hafler JP, Pohl-Koppe A. The cellular immune response to *Bordetella pertussis* in two children with whooping cough. Eur J Med Res. 1998 Nov 17;3(11):523-6.
4. Health Council. Vaccinatie tegen kinkhoest. Den Haag: Gezondheidsraad. 2004. publicatie nr. 2004/04.
5. [Hedenskog S, Bjorksten B, Blennow M, Granstrom G, Granstrom M.](http://www.ncbi.nlm.nih.gov/entrez/query.fcgi?cmd=Retrieve&db=pubmed&dopt=Abstract&list_uids=2759713) Immunoglobulin E response to pertussis toxin in whooping cough and after immunization with a whole-cell and an acellular pertussis vaccine. Int Arch Allergy Appl Immunol. 1989;89(2-3):156-61.
6. Hellwig SM, van Spriel AB, Schellekens JF, Mooi FR, van de Winkel JG. Immunoglobulin A-mediated protection against *Bordetella pertussis* infection. Infect Immun. 2001 Aug;69(8):4846-50
7. Hewlett-EL; Halperin-SA. Serological correlates of immunity to *Bordetella pertussis*. Vaccine, 1998, Vol. 16: 1899-1900

15. von Hunolstein C., Aggerbeck H., Andrews N., Berbers G., Fievet-Groyne F., Maple P.A.C., Ölander R.-M. Raux M., & Tischer A. European Sero-Epidemiology Network: standardization of the results of diphtheria antitoxin assays. Vaccine 2000, 18, 3387-3296.

16. Kim YS, Kwon KS, Kim DK, Choi IW, Lee HK. Inhibition of murine allergic airway disease

by *Bordetella pertussis*.Immunology. 2004 Aug;112(4):624-30.

17. Labadie-JL; Sundermann LC; Rümke-HC. Multi-center study of the simultaneous administration of DPT-IPV and Hib PRP-T vaccines. RIVM Report 124001003, Bilthoven, 1996.

18. Lacombe K, A Yama, K Simondona, S Pinchinata, F Simondona. Risk factors for acellular and whole-cell pertussis vaccine failure in Senegalese children. Vaccine 2004 623-8

19. Leef M, Elkins KL, Barbic J, Shahin RD. Protective immunity to *Bordetella pertussis* requires both B cells and CD4(+) T cells for key functions other than specific antibody production. J Exp Med. 2000 Jun 5;191(11):1841-52.

20. Mariani M., Luzzi E., Proietti D., Mancianti S., Casini D., Costantino P., van Gageldonk P. & Berbers G. A competitive enzyme-linked immunosorbent assay for measuring the levels of serum antibody to *Haemophilus influenzae* type b. Clin. Diagn. Lab. Immun., 1998, 5, 667-674.

21. Mascart F, Verscheure V, Malfroot A, et al. *Bordetella pertussis* infection in 2-month-old infants promotes type 1 T cell responses. J immunol 2003;170:1504-9

22. Manclark-CR; Meade-BD; Burstyn-DG. Serological response to *Bordetella pertussis*. Manual Clinical Immunology (third edition), 1986, 388-394.

23. Meade-BD; Deforest-A; Edwards-KM et al. Description and evaluation of serologic assays used in a multicenter trial of acellular pertussis vaccines. Pediatrics, 1995, Vol. 86: 570-575, supplement.

24. McGuirk P and KHG. Mills. Direct anti-inflammatory effect of a bacterial virulence factor: IL-10 dependent suppression of IL-12 production by filamentous hemagglutinin from *Bordetella pertussis*. Eur. J. Immunol. 2000, 30: 415–422.

25. J McVernon, N Andrews, M P E Slack, M E Ramsay. Risk of vaccine failure after Haemophilus influenzae type b (Hib) combination vaccines with acellular pertussis. Lancet 2003; 361: 1521–23

26. Mills KH. Immunity to *Bordetella pertussis*. Microbes Infect. 2001 Jul;3(8):655-77.

27. [Odelram H, Granstrom M, Hedenskog S, Duchen K, Bjorksten B.](http://www.ncbi.nlm.nih.gov/entrez/query.fcgi?cmd=Retrieve&db=pubmed&dopt=Abstract&list_uids=8087191) Immunoglobulin E and G

responses to pertussis toxin after booster immunization in relation to atopy, local reactions and aluminium content of the vaccines. Pediatr Allergy Immunol. 1994 May;5(2):118-23.

28. Olin P, Rasmussen F, Gustafsson L, Hallander HO, Heijbel H. Randomized controlled trial of two-component, three-component, and five-component acellular pertussis vaccines compared with whole-cell pertussis vaccine. Ad Hoc Group for the Study of Pertussis Vaccines. Lancet. 1997 Nov 29;350(9091):1569-77.

29. Renz, H, E von Mutius, S Illi, F Wolkers, T Hirsch, SK Weiland. Th1/Th2 response profiles differ between atopic children in eastern and western Germany. J Allergy Clin Immunol 2002;109:338-42

30. Rowe J, ST Yerkovich, P Richmond, D Suriyaarachchi, E Fisher, L Feddema, R Loh, PD Sly, and PG Holt. Th2-Associated Local Reactions to the Acellular Diphtheria-Tetanus-Pertussis Vaccine in 4- to 6-Year-Old Children. Infect. Immun. 2005 73: 8130-8135.

31. Ryan M, Murphy G, Ryan E, Nillson L, hackley F, Gothefors L et al. Distinct T cell subtypes induced with whole cell and acellular pertusssis vaccines in children Immunology 2003;170:1504-9

32. Ryan M, Murphy G, Ryan E, Nilsson L, Shackley F, Gothefors L, Oymar K, Miller E, Storsaeter J, Mills KH. Distinct T-cell subtypes induced with whole cell and acellular pertussis vaccines in children. Immunology. 1998 Jan;93(1):1-10.

33. Simondon F, Preziosi MP, Yam A, Kane CT, Chabirand L, Iteman I, Sanden G, Mboup S, Hoffenbach A, Knudsen K, Guiso N, Wassilak S, Cadoz M. A randomized double-blind trial comparing a two-component acellular to a whole-cell pertussis vaccine in Senegal. Vaccine. 1997 Oct;15(15):1606-12.

34. Storsaeter J, Hallander HO, Gustafsson L, Olin P. Levels of anti-pertussis antibodies related to protection after household exposure to *Bordetella pertussis*. Vaccine. 1998 Dec;16(20):1907-16.

35. Weiss AA, Mobberley PS, Fernandez RC, Mink CM. Characterization of human bactericidal antibodies to *Bordetella pertussis*. Infect Immun. 1999 Mar;67(3):1424-31.

36. Weiss-A. Mucosal Immune Defenses and the Response of *Bordetella pertussis*. ASM News, 1996, 63: 22-28.

37. [Zepp F, Knuf M, Habermehl P, Schmitt HJ, Meyer C, Clemens R, Slaoui M.](http://www.ncbi.nlm.nih.gov/entrez/query.fcgi?cmd=Retrieve&db=pubmed&dopt=Abstract&list_uids=9272364) Cell-mediated immunity after pertussis vaccination and after natural infection. Dev Biol Stand. 1997;89:307-14.

38. SC de Greeff, FR Mooi, JFP Schellekens, HE de Melker. Impact of acellular pertussis

preschool booster vaccination on disease burden of pertussis in the Netherlands. PIDJ 2008

39. P.G.M. van Gageldonk, F.G. van Schaijk, F.R. van der Klis, G.A.M. Berbers. Development
 and validation of a multiplex immunoassay for simultaneous determination of serum antibodies

to *Bordetella pertussis*, diphteria and tetanus. J.of Imm.Methods, 2008;Mar26

40. [www.euvac.net/graphics/euvac/images/vaccination/pertussis.gif](http://www.euvac.net/graphics/euvac/images/vaccination/pertussis.gif)
